# Evolutionary Insight into Fatal Human Coronaviruses (hCoVs) with a Focus on Circulating SARS-CoV-2 Variants Under Monitoring (VUMs)

**DOI:** 10.3390/biomedicines13102450

**Published:** 2025-10-08

**Authors:** Mohammad Asrar Izhari, Fahad Alghamdi, Essa Ajmi Alodeani, Ahmad A. Salem, Ahamad H. A. Almontasheri, Daifallah M. M. Dardari, Mansour A. A. Hadadi, Ahmed R. A. Gosady, Wael A. Alghamdi, Bakheet A. Alzahrani, Bandar M. A. Alzahrani

**Affiliations:** 1 Department of Laboratory Medicine, Faculty of Applied Medical Sciences, Al-Baha University, Al-Baha 65528, Saudi Arabia; 2 Laboratory Department, Prince Meshari Bin Saud Hospital, Al-Baha 65639, Saudi Arabia; 3 Department of Dermatology, Al-Kharj Military Hospital, Al-Kharj 11361, Saudi Arabia; 4 Sabt Al Alaya General Hospital, Sabt Al Alayah 67512, Saudi Arabia; 5 Ibn Sina Hospital for Extended Care, Makkah 24211, Saudi Arabia; 6 Laboratory Department, Baish General Hospital, Jazan 87597, Saudi Arabia; 7 Department of Microbiology, Specialized Hospital, Jazan 87597, Saudi Arabia; 8 Laboratory Department, King Fahad Hospital Al-Baha, AI-Baha 65732, Saudi Arabia

**Keywords:** hCoVs, VUM, mutation, evolution, lineage, variants

## Abstract

The breach of an interspecies barrier by RNA viruses has facilitated the emergence of lethal hCoVs, particularly SARS-CoV-2, resulting in significant socioeconomic setbacks and public health risks globally in recent years. Moreover, the high evolutionary plasticity of hCoVs has led to the continuous emergence of diverse variants, complicating clinical management and public health responses. Studying the evolutionary trajectory of hCoVs, which provides a molecular roadmap for understanding viruses’ adaptation, tissue tropism, spread, virulence, and immune evasion, is crucial for addressing the challenges of zoonotic spillover of viruses. Tracing the evolutionary trajectory of lethal hCoVs provides essential genomic insights required for risk stratification, variant/sub-variant classification, preparedness for outbreaks and pandemics, and the identification of critical viral elements for vaccine and therapeutic development. Therefore, this review examines the evolutionary landscape of the three known lethal hCoVs, presenting a focused narrative on SARS-CoV-2 variants under monitoring (VUMs) as of May 2025. Using advanced bioinformatics approaches and data visualization, the review highlights key spike protein substitutions, particularly within the receptor-binding domain (RBD), which drive transmissibility, immune escape, and potential resistance to therapeutics. The article highlights the importance of real-time genomic surveillance and intervention strategies in mitigating emerging variant/sub-variant risks within the ongoing COVID-19 landscape.

## 1. Introduction

Evolutionary dynamics refer to the changes that occur in biological entities driven by mutations, natural selection, and genetic variations [1]. In recent decades, highly virulent hCoVs, such as the severe acute respiratory syndrome coronavirus (SARS-CoV) [2], have evolved first. Later, Middle East respiratory syndrome (MERS-CoV) [2] emerged. Subsequently, SARS-CoV-2 [3] evolved, causing severe health concerns worldwide. These viruses have potentially caused significant human mortality and posed serious global public health challenges [4,5]. With widespread travel and global trade, modern life facilitates the rapid spread of viruses, posing additional health risks worldwide [6]. However, there are a few less lethal hCoVs (hCoV/229E exploiting aminopeptidase N (hAPN) receptor, hCoV/NL63-interacting with ACE2, hCoV/OC43-interacting with (9-O-acetylated sialic acid), and hCoV/HKU1 leveraging 9-O-acetylated sialic acid) identified to cause mild symptoms [7]. Kistler and Bedford (2021) highlighted how adaptive evolution (genetic drift) may enable hCoV/229E and hCoV/OC43 viruses to escape immune detection, raising the possibility of increased transmissibility or disease severity in the future [8]. Additionally, Ye et al. (2023) demonstrated through whole-genome sequencing and phylogenetic analyses in a multicenter study that novel lineages continue to emerge for HCoV-229E, HCoV-NL63, and HCoV-OC43, indicating that the ongoing genetic divergence suggests the likelihood of more variants potentially altering viral behavior and risk over time [9].

Seasonal hCoVs such as 229E, NL63, OC43, and HKU1 circulate globally with clear patterns of seasonality, particularly in temperate regions, often causing respiratory illnesses during the winter months. Their predictable seasonal peaks significantly contribute to the overall burden on healthcare systems, influencing resource allocation and underscoring the need for effective public health surveillance. Evolving viral traits in these hCoVs further elevate the importance of continual monitoring, as they may inform our understanding of spillover risks and preparedness for more virulent coronaviruses [10]. The high mortality due to these fatal hCoVs and their variants/subvariants posed dual challenges: the public health risk of contracting these infections and the systemic burden imposed on healthcare infrastructure, encompassing physicians and allied health professionals [11,12]. Although definitive therapy is currently lacking, significant advancements in outcomes have been achieved using supportive treatment [13,14]. Moreover, a vaccine against MERS-CoV is not yet available [14]. The success of the vaccine drive using Moderna and Pfizer–BioNTech (mRNA vaccines) in combating infections with SARS-CoV-2 has been significantly underscored globally [15,16]. However, the vaccine’s potency with currently circulating SARS-CoV-2 variants/subvariants [15] and adverse effects remain under discussion [17,18,19]. Close surveillance and monitoring of circulating variants/subvariants capable of evading mechanisms remain a primary concern to physicians and health authorities worldwide [20]. Moreover, precise knowledge about the genetic fingerprints (especially those of the spike protein) of the currently circulating variants is paramount for facilitating the development of effective vaccines and therapeutics [21,22].

**Figure 1:**
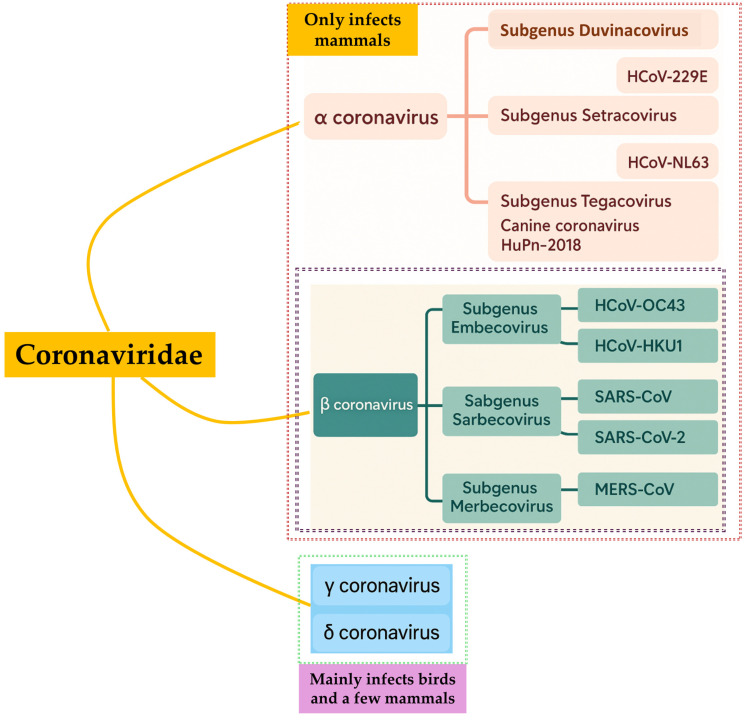
Representation of the relatedness-based 10th ICTV classification report [7].

This study primarily aims to investigate the mutational dynamics of SARS-CoV to SARS-CoV-2 VUMs as of May 2025, with a specific focus on the evolutionary significance of mutations in the spike protein. We aim to understand how changes in the RBD and its receptor-binding motif (RBM) contribute to viral adaptation, the emergence of new variants, and the virus’s ability to evade immune surveillance. This research is essential for improving the preparedness of the scientific community in combating future pandemics, particularly in the development of vaccines and therapeutic interventions targeting key viral components. Figure 1 illustrates an evolutionary outline of different coronaviruses [7]. hCoVs can be categorized into four (*n* = 4) genera: Alpha/α, Beta/β (divided into different clades), Gamm/γ, and Delta/δ. Beta/β-hCoVs comprise SARS-CoV-2 in addition to MERS-CoV. This review summarizes the evolutionary trajectory of three lethal hCoVs (from the origin of the first lethal hCoVs to SARS-CoV-2 VUMs in circulation as of May 2025). It also highlights critical spike mutations and their clinical implications using bioinformatics strategies, which are pivotal for strategizing enhancements in diagnostic accuracy, maintaining vaccine protection, and developing potential targeted therapeutics.

## 2. SARS-CoV

### 2.1. Emergence as an Alarm to a Lurking Pandemic (SARS-CoV-2 Pandemic)

SARS cases originally appeared in China’s Guangdong (at the end of 2002) and, within just a few months, swiftly spread across 25 countries spanning five continents [23]. Shortly after the initial outbreak of SARS, the coronavirus was identified as the etiology of the SARS outbreak [24,25], based on the determination of complete genomic sequences (RefSeq) followed by evolutionary studies [24,25,26]. Identifying the raccoon dogs as well as palm civets (in the Guangdong market for live animals) as potential sources of the SARS etiological agent (SARS-CoV) delivered the initial hints of a zoonotic spillover (animal-to-human spillover) [27]. Later, various other SARS-like-CoVs harboring in horseshoe bats (*Rhinolophus* spp.) were detected and documented, suggesting that bats might serve as natural reservoirs of SARS-CoV and many other closely associated hCoVs [28,29,30,31]. Although no new SARS-CoV cases were presenting at that point, the recurrence of SARS-CoV remained uncertain. It was hypothesized that SARS-CoV was surviving in its natural source, which may result in an episode of recurrence at any point in time.

### 2.2. Genomic Organization

SARS-CoV contains a peplomer-bearing envelope with a genome similar to mRNA (approximately 29,700 nucleotides in size). Fourteen open reading frames (ORFs) comprise the genome, which corresponds to twenty-eight proteins. These proteins are categorized into three distinct groups. The first group comprised a large polyprotein group (*n* = 02). The second category included structural proteins (SP), comprising S, E, M, and N (*n* = 4).

Moreover, the third group encompassed eight (*n* = 8) accessory proteins (AP). P1a and P1ab were identified as the two larger polyproteins that underwent the cleavage process to produce 16 viral non-structural-proteins (NSPs). The NSPs were abbreviated as nsp1 to nsp16. Four SPs are determinants of infectivity (viral entry and assembly). The APs are not significant in viral replication; however, their role is pivotal in other equally important processes, such as viral assembly, virulence, and pathogenesis [32,33,34,35].

### 2.3. Evolution in SARS-CoV

High variability at both genomic and antigenic levels is consistently associated with all RNA-genome-containing viruses, including SARS-CoV, and this variation fuels viral evolution. One of the most critical metrics for quantifying evolutionary dynamics is the ω (dN/dS) ratio, which provides information about whether a gene is under selection pressure. It helps distinguish between:

Neutral selection with ω = 1 signifies the random evolution, suggesting the mutation is neither advantageous nor deleterious.

Negative (purifying) selection with ω < 1 signifies that the deleterious mutations are omitted to conserve the function of the gene.

Positive (diversifying) selection with ω > 1 signifies that beneficial mutations in genes for immune evasion and adaptation are favored [36,37].

In one of the earlier studies, ω > 1 for the SARS-CoV S-protein was observed during the SARS epidemic, underscoring a higher rate of nonsynonymous substitutions under positive selective pressure [38,39,40]. Moreover, the estimated ω for the spike gene revealed positive selection, underscoring its role in SARS-CoV adaptation [41]. In contrast, purifying selection was observed in most of the structural and nonstructural genes, elucidating the functional constraints on replication [41,42]. However, there is insufficient evidence to achieve ω > 1 among the replicase or Aps, which might be of great significance, thereby enabling SARS-CoV to adapt to a new host. With a motto of attempting to reach the bottom of the SARS-CoV’s adaptive evolutionary landscape, the more refined model (branch site) was executed to study and gain insights into the selective pressures that could foster a notable action on some of the pivotal SARS-CoV essential functional proteins.

### 2.4. Evolution in Spike (S)-Protein

**Figure 2:**
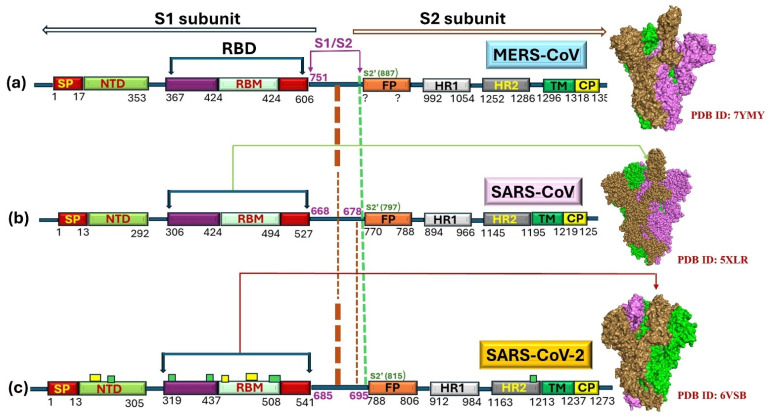
Comparative illustration of S-protein of hCovs (MERS-CoV and SARS-CoV/CoV-2). (**a**) MERS-CoV’s spike (prefusion RBD up conformation represented by 3D density map), (**b**) SARS-CoV’s spike (RBD up conformation depicted by 3D density map), and (**c**) SARS-CoV-2’s spike (prefusion RBD up conformation represented by 3D density map). FP = fusion peptide, RBM = receptor-binding motif (facilitate specific interaction with host receptor), RBD = receptor-binding domain (interaction with host receptor), SP = signal peptide, HR1 = heptad repeat 1 (involved in fusion process), HR2 = heptad repeat 2 (involved in fusion process), TM = transmembrane (ensure anchorage and structural integrity of spike in viral membrane facilitating viral entry), NTD = N-terminal domain, CP = cytoplasmic (anchoring and embedding of spike in viral envelop), S1/S2 (subunits) represents the furin-cleavage-sites which is the junction point of S1-subunit and spike’s S2-subunit, and S2′ = S2′-protease (furin-cleavage-site). Protease-cleaving-spots/sites (S1/S2-junction furin-like cleavage-spots/sites represented in red dashed lines) and protease cleavage sites (S2′ furin-like cleavage site represented in light green dashed line). The varying weight of the dashed lines indicates the different cleavage propensity. MERS-CoV is deficient in the 2nd S1/S2-junction cleavage-spot/site. Yellow and greenish rectangles depicted in the SARS-CoV-2 protein illustrate minimum and maximum significant conservative regions, accordingly [47,48,49].

The S-protein, a pivotal structural protein of hCoVs, including SARS-CoV, drives infectivity by engaging with the ACE2 receptor via its RBD, which is embedded in the S1 subunit. The receptor-binding motif (RBM) binds to the ACE-2 receptor, facilitating viral penetration [43]. The S-protein comprises S1 and S2 subunits and modifies itself by undergoing cleavage. A schematic representation of the S-protein of MERS-CoV, SARS-CoV, and SARS-CoV-2 was created using PyMOL for visualizing the protein structures (Figure 2). The spike protein conformations were depicted based on 3D density maps from the PDB structures (7YMJ, 5XLR, and 6VSB). The protein domains and receptor-binding domain (RBD), including the receptor-binding motif (RBM) and fusion peptide (FP), were highlighted to show the structural differences between the variants. Figure 2 illustrates the comparative aspects of the S-protein of hCovs (MERS-CoV and SARS-CoV/CoV-2). The S1/S2 junction is the site where cleavage occurs. This junction is recognized as the cleavage site in the coronavirus of mammalian or avian origin, as illustrated in Figure 2b [44]. A highly divergent S1 subunit possessing RBD where receptor-binding motif (RBM) remains embedded and a comparatively more conserved S2 domain, each made up of two heptad repeats (HR) domains, as depicted in Figure 2b [45]. It was observed from various detailed studies that, during an outbreak of the SARS infection, distinguishable positive selection was noted for gene S of SARS, prominently during the initial and mid-phase of the outbreak [38,39,40]. Only a small proportion of sites within the S-protein exhibited strong positive selection during the 2002–2003 SARS epidemic for the phases mentioned above. Conversely, positive selection was noticed for the isolates from the late phase, indicating that the S-protein attains stability again after the early and middle stages of evolution. Reports suggested that SARS-CoVs emerged initially in bats, which later spilled over into civets. The two SARS outbreaks that occurred recently, with a gap of one year in between, were not due to animal-to-human transmission [27,38]. Most of the divergence was observed in the S-gene of SARS of two species (bats and civets), indicating the probability of a close association between SARS-CoVs in bats or of another unidentified animal [46].

### 2.5. Natural Selection

Natural selection favors the reduction of deleterious mutations over the promotion of advantageous mutations. High mutation rates, relaxed selective constraints (losing functional significance), or natural selection (positive, negative, or balancing selection) could be possible factors for a gene to be significantly divergent [50]. Genes encoding proteins involved in the viral life cycle process are significant. Once a virus has successfully entered a cell, the proteins engaged at any stage of its life cycle may undergo adaptive evolution. Adaptation and gene diversity often result from two important evolutionary mechanisms–mutations and recombination. Putative recombination was observed following a thorough comparative genomic analysis of SARS-CoV and other hCoVs [51,52,53]; nonetheless, no recombination was observed when only SARS-CoV sequences were analyzed [31,40]. Phylogenetic analytical studies, primarily based on computationally driven split decomposition and/or maximum likelihood methods, have revealed evidence of putative recombination events, which makes the evidence of the RNA virus’s recombination issue controversial. Hence, one must be cautious when drawing inferences and reaching conclusions based on analyses of putative recombination events. Adequate maintenance of the recombinant during evolution (post-recombination), stability and reproducibility of the recombination in viable virus clonal populations, and a single amplicon generated by PCR happening in a molecule of DNA should be capable of proving the occurrence of the recombinant crossover are the pivotal criteria for confirming transmission of a clone of recombinant viruses emerged from natural recombination [51].

### 2.6. Evolutionary Dynamics of SARS-CoV

Sequential adaptation of SARS-CoVs to humans was observed [52]. During the initial and middle phases of the outbreaks in palm civets and humans, the S-protein underwent robust positive selection, facilitating viral entry into target cells [53]. Contrastingly, the positive selection for replicase protein was observed only in cases of human infection; however, it was not noticed in the case of palm civets, suggesting that palm civets can serve as an optimal host for studying the replication process in SARS-CoV [54]. Positive selection was observed for a protein that contributed to viral assembly (maturation) and exit (release) during the middle and later phases of the epidemic. These data reveal that, under certain conditions, such as an infection outbreak and a narrow period, evidence supporting SARS as a zoonotic origin suggests that specific conserved amino acids play a pivotal role in adapting to a vast range of hosts.

Understanding the evolutionary dynamics of SARS-CoV provides a valuable foundation for examining other pathogenic human coronaviruses, such as MERS-CoV. Both viruses contain an RNA genome and exhibit high genetic flexibility, which facilitates rapid adaptation to host immune responses and environmental pressures. The role of spike protein mutations and alterations in the receptor-binding domain in SARS-CoV illustrates mechanisms that similarly influence the emergence and host specificity of MERS-CoV. However, while SARS-CoV spread predominantly through human-to-human transmission during outbreaks, MERS-CoV is primarily maintained in its animal reservoir, dromedary camels, with sporadic human infections. The following section describes common evolutionary strategies and unique transmission patterns of MERS-CoV.

## 3. MERS-CoV

### 3.1. Emergence

According to reports generated by the WHO as of January 2016, MERS-CoV has affected 1638 human infections, resulting in 587 deaths [55]. According to the WHO, MERS, caused by a coronavirus, is a highly lethal disease and a significant threat to the global population [56,57,58]. Since the initial identification of MERS-CoV in the Kingdom of Saudi Arabia (KSA) in 2012, a cumulative total of 2627 laboratory-confirmed cases has been documented globally, accompanied by 946 fatalities, corresponding to an overall case fatality rate (CFR) of approximately 36% (WHO, 2025). These cases span 27 countries across all 6 WHO regions; however, the vast majority (2218 cases, or 84%) have been reported from KSA, including the most recent notifications from Riyadh. Notably, since 2019, no confirmed human MERS-CoV infections have been reported outside the Middle East, indicating that the virus remains primarily geographically constrained to its region of origin (https://www.who.int/emergencies/disease-outbreak-news/item/2025-DON569, accessed on 24 September 2025).

MERS-CoV contains RNA (ss) as the genetic material [59]. A high mortality rate is observed due to MERS-CoV infection in countries such as the Gulf region, the Korean region, and the European region [60]. The virus exhibits taxonomical similarity with SARS-CoV and is associated with severe respiratory diseases [61]. In 2012, the first human case affected by this infection was reported in Saudi Arabia [62]. A similar infection was registered in Egypt, where the virus was isolated from the patient’s lungs. A similar report was recorded from the Kingdom of Saudi Arabia (KSA), Egypt, and several countries, including Indonesia, the United Arab Emirates (UAE), Qatar, China, Thailand, Korea, the United Kingdom (UK), and the United States of America (USA) [58,63]. MERS-CoV was further characterized in many provinces of KSA and various other geographical locations [64]. Korea reported 185 MERS-CoV cases till today, 185 cases of which 36 proved to be fatal [65]. The extensive research carried out and documented on MERS-CoV infection suggested that the “camels” were the natural reservoirs of infection [66]. It was observed that the people suffering from MERS in the initial stages were exposed to camels either through contact or by consuming camel milk. Humans acquire the infection from camels [67]. This infection is transmitted from affected individuals to healthcare personnel via contact with the infection-prone areas and droplet infection [59,68]. To date, no precise mode of treatment or vaccination therapies has been developed for MERS-CoV [69]. To prevent the spread of MERS-CoV, it is advisable to refrain from contacting camels or affected individuals and to practice appropriate sanitation and hygiene [70].

### 3.2. Diversity of MERS-CoV

A glimpse of the diversity among dromedary camels was studied by Sabir et al. [71]. Of the total number of source animals, i.e., camels, under their study, they observed that approximately twenty-five percent (25.3%) of camels were detected positive for different species of hCoVs. The detected hCoVs encompassed b1-HKU23-CoV (Beta-CoV clade), camelid/CoV (Alpha-CoV), and MERS-CoV. The camelid-CoV most frequently coinfected the cell with MERS-CoV. Moreover, dromedary camels, European hedgehogs, and two species of bats were the entities in which MERS-CoV appeared. It was proven that animals like bats and hedgehogs were the source of origin of camel and human MERS-CoVs [2].

### 3.3. Evolution of S-Protein

The early requisite steps in eliciting the infection are determined by the S-protein (a crucial structural protein), a principal component of the viral structure, which, upon interaction with the appropriate receptor, defines the tissue tropism [72]. The S-protein encompasses two functionally diverse domains: S1 and S2. The S1 region, in the RBD, remains embedded. S1 region also encompasses the N-terminal domain (NTD). However, the S2-region is constituted of a transmembrane region. S2 region comprised a fusion peptide (FP), in addition to the heptad-repeats (HRs). HRs were abbreviated as HR1 and HR2, as represented in Figure 2a [73]. Positive selection and genetic recombination aided the S-gene variability, which caused an increase in the viral (MERS-CoV and related-Beta-CoVs) sequence number. Additionally, the initial and middle phases of rapid intra-spike recombinations were determined and marked [74,75]. It was revealed that the S-gene with breakpoints during the recombination events existed in Saudi camels, which led to the development of several lineages of the MERS-CoV, which later gradually spread to the regions of South Korea [76].

A wide range of MERS-CoV and phylogenetically close hCoVs’ adaptive variants were generated because of the S-gene evolutionary process (positive selection) in MERS-CoV [77]. However, the RBD of the S1 region was not subjected to positive selection, which was contradictory to the expected results. The domains that were susceptible to adaptive substitutions were the regions surrounding the heptad repeats of the S2 region. Most adaptive substitutions/mutations were determined in the area encompassing the heptad repeats, which are of prime importance for facilitating the entry of the virus into the host cell [77,78]. Variations in these heptad repeats were found to affect host or tissue tropism [79,80,81].

Through the MERS epidemic in South Korea, it was spotted that the point mutations occurring in the RBD were the main reason behind the rapid spread of MERS, but the virus displayed comparatively lower affinity for the cellular receptor-cluster of differentiation 26 (CD26)/dipeptidyl peptidase-4 (DPP4) [74]. Since the RBD region possesses diverse immune epitopes, the observations guide us towards the probability that the virus is advancing to circumvent the binding of the specific antibodies against it [74]. If this hypothesis is to be accepted, then the series of changes that occur in the viral genetic make-up due to modulations of the heptad regions would have provided the initial phases of MERS-CoV adaptation to humans. Positive selection in MERS-CoV and other co-related lineages targets the structural domains and the nonstructural components, especially the open reading frame 1a (ORF1a) [77,82]. nsp3, a protein with diverse functionality, behaves like a viral-protease and participates in suppressing the response generated by interferons through its deubiquitinating and deI-SGylating activities, which witnesses most of the adaptive changes [83]. Moreover, the evolutionary events were seen among MERS-CoV of both human and camel origin as a result of selection in nsp3 [77,82]. As no noticeable selective events were found to be related to camel–human transmission, even though a few modifications (R911C) in nsp3 were noted due to positive selection, it suggested that the adaptations of viruses specific to humans represented the underlying pressure [82].

Human coronaviruses (hCoVs), including seasonal strains such as HCoV-OC43 and HCoV-NL63, as well as more pathogenic variants like SARS-CoV and MERS-CoV, exhibit distinct evolutionary trajectories and mutational behaviors. These viruses are genetically diverse, and their capacity for adaptation through mutations plays a crucial role in their transmission dynamics and pathogenicity [84]. SARS-CoV-2, while genetically related to its predecessors, SARS-CoV and MERS-CoV, has demonstrated a more pronounced mutational burden, significantly impacting its transmissibility and capacity for immune evasion [85]. Building on the insights gained from earlier hCoVs, the subsequent section explores the mutational landscape of SARS-CoV-2, with a particular focus on the spike protein (S-protein). RBD of this protein, which mediates viral entry via the ACE2 receptor, has undergone multiple mutations that enhance its affinity for ACE2, thereby augmenting the virus’s infectivity and contributing to the emergence of new variants with varying levels of transmissibility and immune escape potential.

## 4. SARS-CoV-2’s Story of Emergence

SARS-CoV-2 triggered the first human infection in the Wuhan region of China and later became a reason for the COVID-19 pandemic [86]. Biologically and symptomatically, it exhibits genetic proximity (greater similarity) to SARS-CoV; therefore, it is termed SARS-CoV-2 [87]. The similarity in the sequences of the genomic regions of SARS-CoV-2 with other beta coronaviruses (bat SARS-like coronavirus, SARS-CoV, as well as MERS-CoV) has been highlighted in Figure 1 [88]. The timeline for the evolution of these lethal hCoVs was observed as SARS-CoV (in 2002) from China, MERS-CoV from KSA (in 2012). Later, SARS-CoV-2 (in 2019) again emerged from China [89,90,91]. Initially, epidemiological investigation revealed possible evolution (spillover) of SARS-CoV-2 as a distinct divergent hCoV from intermediate hosts (bats, pangolins, snakes). Moreover, bats have been reported as a primary pathogen reservoir and natural niche for the SARS-CoV-2 virus [92,93,94,95]. While bats are considered the primary reservoir of SARS-CoV-2, the exact intermediate host responsible for the zoonotic spillover to humans remains uncertain. Pangolins, civets, and raccoon dogs have been proposed as potential candidates, but no definitive species has yet been confirmed [96].

### 4.1. Mutational Burden in Spike Protein Fuels Variant Generation and Alters Evolutionary Dynamics

Gaining a deeper understanding of the evolutionary landscape and trajectory of SARS-CoV-2 is crucial for comprehending its infectivity, degree of pathogenesis, and extent of virulence. Adaptation to the environment (immune response and selective pressure) and fidelity errors are the key drivers of mutagenesis, which fuels the generation of variants/sub-variants to change the SARS-CoV-2’s evolutionary landscape [97,98].

**Figure 3:**
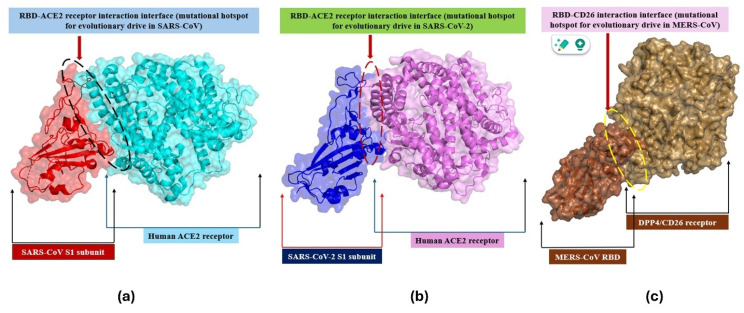
3D shadow comparative representation of the catalytic spots for lethal hCoV variant evolution. (**a**) SARS-CoV’s RBD-human ACE2 receptor interface, (**b**) representation of SARS-CoV-2’s RBD-human-ACE2-receptor conformational interface, and (**c**) illustrations of MERS-CoV’s RBD-human CD26/DPP4 receptor interface.

The most significant mutational site in SARS-CoV/CoV-2 is the spike’s RBD, as illustrated in Figure 3a,b [99,100]. However, the MERS-CoV’s RBD interacts with another type of cellular Receptor (CD26/DPP4), as depicted in Figure 3c [101]. Therefore, the mutational burden in the RBD may alter (strengthen) the binding potential of the RBD with cellular receptors, leading to the generation of highly infectious variants [102,103]. Events of specific single-nucleotide polymorphisms (SNPs)/mutations favor the process of natural selection in which infectivity-enhancing SNPs outperform infectivity-diminishing SNPs [1]. Thus, occasionally, such SNPs/mutations foster the ability to diminish the vaccine efficacy, evasion ability, and greater transmissibility to the evolving variants [104]. One of the most dominant global variants evolved just because of a single SNP (D614G) with enhanced infectivity, highlighting the significance of even a single SNP in the hot spot in the evolutionary process [1].

One of the major driving factors for generating mutational burden is the lower fidelity of RNA polymerase (prone to transcriptional errors). The mutation (substitution) rate in the SARS-CoV-2’s complete genome was reported as 6.7 × 10^−4^ mutations or substitutions/site per annum. However, that in the spike gene (S-gene) was recorded as 8.1 × 10^−4^ mutation (substitutions)/site per annum. It was also observed that the substitution/mutation rate in SARS-CoV-2 was rather lower in spite of having an RNA polymerase [105]. Rapid evolution and generating a vast number of variants/sub-variants globally, despite a relatively lower nucleotide mutation rate, could be explained by the prolonged pandemic period and spread to a larger global population [1,106]. In addition to mutation, genetic recombination during coinfection of a cell with two or more different variants contributed to the generation of a vast number of variants, suggesting that the mutation and/or recombination in the hot spot in the spike was the critical driving factor for shaping the evolutionary landscape for the SARS-CoV-2 [107].

### 4.2. Evolutionary Progression of SARS-CoV-2 Lineages

**Figure 4:**
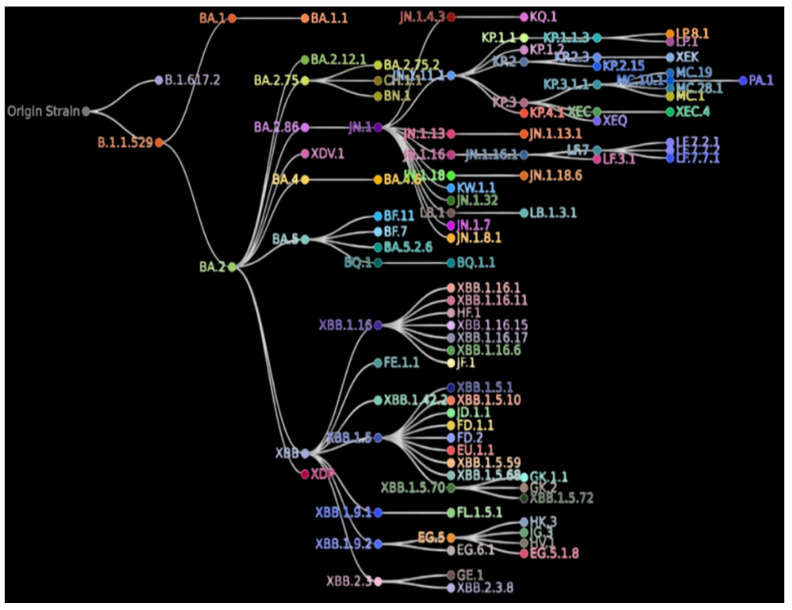
Relation between the Pango lineages on COVID-19 Data Trackers. Each node represents a SARS-CoV-2 lineage/sub-lineage descending from its previous lineage. The image is a modified version of the image retrieved from the CDC COVID Data Tracker [109], URL: https://www.cdc.gov/covid/php/variants/variants-and-genomic-surveillance.html (accessed on 12 June 2025).

Clinical management of the COVID-19 disease is further complicated by the evolution of variants/sub-variants, with varying severity and mortality. Moreover, the virulent lineages pose enormous challenges to the efficiency of the preventive and therapeutic countermeasures [108]. Figure 4 illustrates the evolutionary trajectory of the SARS-CoV-2-pango-lineages.

The rapid and continuously evolving distinct SARS-CoV-2 lineages/sub-lineages (categorized mostly according to the spike mutations) during the COVID-19 pandemic led to the death of >7 million individuals [110]. The spike protein, mediating viral entry, was determined as a pivotal determinant of infectivity, transmissibility, pathogenicity, and tissue tropism [111,112]. The central role of S-protein in evolution has been underscored in several studies, particularly regarding adaptation to human hosts and immune evasion, because it is the critical target for immunological response triggered in vaccinated or infected individuals [112]. Therefore, to get comprehensive insights into COVID-19, it is paramount to correlate the variability of S with the evolving phenotypes of the CoV-2.

**Figure 5:**
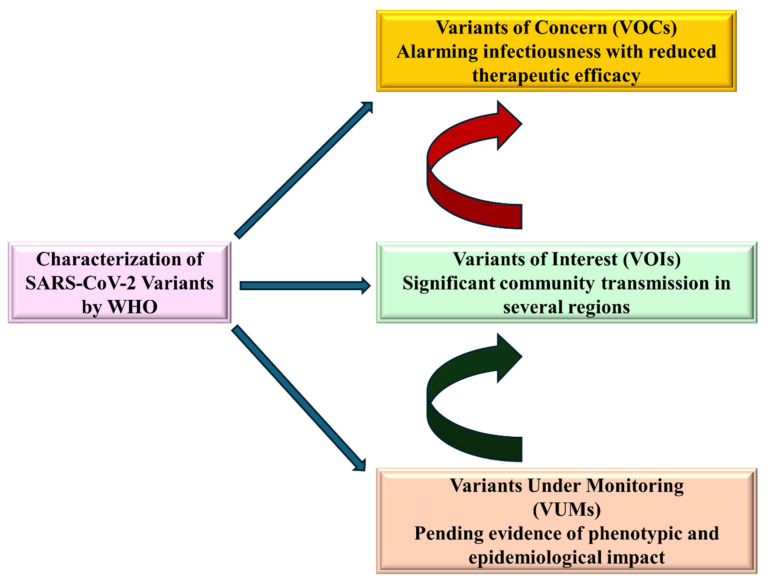
Evolutionary trajectory and categorization of evolving SARS-CoV-2 variants/sub-variants with different medical impacts by the WHO to advance the promising research.

The spike segment, at a polybasic furin cleavage site (FCS), undergoes cleavage (proteolytic maturation) to produce S1 encompassing the RBD and the NTD as well as S2 subunits during biogenesis of SARS-CoV-2 (Figure 2c) [113]. S1-subunit serves as the principal determinant of tissue tropism because RBD undergoes conformational changes to interact effectively with the ACE2 receptor, facilitating the viral entry into the ACE2 receptor-bearing cells, leading to the severe pulmonary sequela and even beyond (extrapulmonary) [112,113,114,115]. Though the SARS-CoV-2 is responsible for the fatality rate lower than that of MERS-CoV, the SARS-CoV-2 potential to acquire mutations, especially in the S region, led to the evolution of several variants of concern (VOC) and variants of interest (VOI) and variants under monitoring (VUMs) in current circulation with varying degrees of human-to-human transmissibility, as illustrated in Figure 5 [116]. High-resolution cryo-EM structures and biophysical studies offer valuable insights into how key mutations in the RBD of the SARS-CoV-2 spike protein enhance ACE2 engagement. Structural analysis of the N501Y mutant complexed with ACE2 revealed that the tyrosine residue at position 501 inserts into a binding pocket near ACE2’s Y41, thereby strengthening receptor interaction without inducing significant conformational changes in the spike protein. However, this mutation increases the affinity of ACE2 for the N501Y mutant, contributing to its enhanced infectivity [117]. Complementary molecular dynamics simulations further revealed that the N501Y mutation fosters a more stable interface by forming sustained hydrogen bonding with ACE2’s Y41 and K353, leading to increased binding affinity (and a prolonged interaction compared to wildtype) [118].

Additionally, simulations of the N501Y mutant show enhanced electrostatic interactions, particularly through a stronger hydrogen bond between RBD T500 and ACE2 D355, contributing about −4 kcal/mol more favorable binding energy than the wildtype [119]. Structural and dynamic studies elucidated how specific spike protein mutations reinforce ACE2 binding, thereby promoting viral transmissibility and adaptation.

Monitoring these variants/sub-variants, and their implications, is significant because they may significantly reduce vaccine protection and pose ongoing public health threats [116,120]. Emerging evidence suggests that immune escape mutations in the SARS-CoV-2 spike protein have direct implications for both vaccine effectiveness and monoclonal antibody (mAb) therapies [121,122]. Alterations in the RBD not only enhance viral affinity for ACE2 but also diminish the binding of neutralizing antibodies generated through vaccination or prior infection, thereby reducing protective immunity [123]. Harvey et al. (2022) described how mutations such as E484K drive reductions in neutralization titers across key variants [111]. Saha et al. (2025) reported that RBD mutations, such as E484K and N501Y, are associated with decreased neutralizing antibody efficacy and immune escape [124]. Tokhanbigli et al. (2025) linked high vaccination rates with selective pressure on SARS-CoV-2 evolution toward immune escape, suggesting a role in breakthrough infections [125]. Moreover, immune escape can compromise the efficacy of monoclonal antibody therapies, as specific mutations disrupt critical epitopes targeted by these treatments, leading to partial or complete resistance. Carabelli et al. (2023) noted that most therapeutic monoclonal antibodies have been evaded by variants [126]. Cox et al. (2023) highlighted that the FDA revoked bamlanivimab’s emergency use due to resistance conferred by L452R and E484K mutations [121]. Roederer et al. (2024) demonstrated that variants continue to evade updated mRNA vaccines, reinforcing the need for alternative strategies [127]. These findings underscore the need for continuous monitoring of emerging variants, rapid adaptation of vaccine formulations, and the development of broadly neutralizing antibodies to ensure sustained protection against evolving viral strains.

Table 1 summarizes critical mutations (in SARS-CoV-2 VOCs or SARS-CoV-2 VOIs) with their potential evolutionary and clinical significance. One of the most recently circulating VOIs is JN.1 (reported from 133 countries), which originated from the parent lineage, BA.2.86, under heavy immunological pressure [128], where JN.1 exhibited poor affinity to the ACE2-receptor and raised evasion when compared with BA.2.86.1 [129]. JN.1 (next-strain clade/24A) is different from the lineage from it has been evolved (BA.2.86) in having the S: L455S mutation [128], which fosters the JN.1 (BA.2.86.1.1)-lineage with high transmissibility, greater evasion potential, and improved fitness [129,130], and approximately four-fold resistance to sera acquired from vaccinated individuals [129,131]. Limited evidence of JN.1 VOIs about additional health risks is currently being evaluated [132]. However, a surge in cases caused by this variant could be anticipated. According to the update as of 5 February 2025, a significant rise in JN.1 prevalence has been observed, but there is no marked change in severity, and the potential of the diagnostic test is still not compromised [133].

### 4.3. Circulating Variants of Monitoring (VUMs)

The most recent evolution of JN. 1 into various sub-strains with lineage-specific substitutions posed significant public health risks and challenges for currently available prevention and diagnostic measures. Several currently circulating sub-lineages of JN.1, categorized as VUMs, are KP.2-sub-lineage (JN.1.11.1.2), KP.3-sub-lineage (JN.1.11.1.2), LB.1, XEC, and LP.8.1 because their prevalence is increasing swiftly (Table 2). Two key spike substitutions, encompassing S: R346T-substitution and S: F456L-mutation, render the KP.2-sub-lineage (FLiRT) variants with greater fitness and the potential to be the dominant strain globally, as the estimated reproduction number (R_e_) was approximately 1.2 times higher compared to JN. 1 [160]. The effective R_e_ and potential to evade immune response necessitate its monitoring and countermeasures to address the potential challenges of this VUM [161]. In addition to the emergence of the JN.1-variant into KP.2-sub-lineage, another major variant, KP.3-sub-lineage (JN.1.11.1.2), has been observed to have evolved from JN.1 [162]. Furthermore, both KP.2-sub-lineage and KP.3-sub-lineage demonstrated a high evasion potential and R_e_ over JN.1 [163]. KP.3, compared to JN. 1, was found to be more adapted (fitted) to enter the lung epithelial cell [164]. KP3 exhibited higher spike cleavage potential than JN.1 and KP.2, highlighting that the RB substitution (L455S and Q493E) can influence the process of S cleavage despite being divergent from the cleavage site (furin cleavage site) (Table 2) [165]. It was observed that the recurrent RBD substitutions (R346T, F456L, and Q493E) could fine-tune the evasion mechanism, along with the replication fitness of emerging KP.2 and KP.3 sub-lineages, highlighting the crucial necessity for updating the countermeasure policy to combat these VUMs effectively [165].

Another JN.1 descendant variant, called KP.3.1.1 (JN.1.11.1.3.1.1/VUM), with a critical RBD substitution (F456L and Q493E) and an S31 deletion in the NTD, emerged by mid-2024 and became predominant worldwide (Table 2) [169]. These RBD substitutions and NTD-deletion foster increased infectivity/transmissibility and immune evasion [174]. KP.3.1.1 exhibits 1.3 times higher resistance to serum neutralization (immune escape) compared to KP.3, highlighting its evasion potential [175]. The antibody in the general Kenyan population was observed to be less effective against KP.3.1.1 compared to other previous variants [176]. The R_e_ estimated for LB.1 (JN.1.9.2.1/VUM) was more than that of the other JN. 1-descendant-VUMs, for instance, KP.2-sub-lineage and sub-lineage-KP.3 [163]. The lineage LB.1 was assessed as being capable of entering lung cells more efficiently [164]. In addition to the S-protein-substitution/mutation (S: F456L, S: R346T, and S: Q493E), a deletion (S: S31del) was acquired to emerge as an LB.1 variant in mid-2024 and spread in several countries (Table 2) [160].

XEC, a VUM with growing prevalence worldwide, demonstrated the relative effective reproduction number (Re), estimated in August 2024 to be 1.13 times greater than that of KP.3.1.1 [160]. XEC showed that Ab-mediated immune evasion contributed by S: T22N (Table 2) [175,177]. A published study on the clinical outcome (disease severity) of XEC is unavailable; however, evidence suggests that XEC infection is increasing markedly, with minimal health risks compared to other circulating Omicron-descendant variants. LP.8.1, a VUM derived from JN.1 descendant KP.1.1.3, emerged with S: S31- deletion, making it different from the XEC variant, while V445R substitution fosters its greater binding affinity to human ACE2, potentially contributing to the variant’s infectivity/transmissibility (Table 2) [175].

LP.8.1 is spreading rapidly and exhibits a comparable immunogenic edge (immune evasion) to XEC. Moreover, LP.8.1-related infections have not been reported to pose higher severity or additional health risks compared to other circulating co-lineages. It has been observed that the JN.1 descendant lineage, JN. 1.18.1, appears to be spreading worldwide [178]. VUMs, SARS-CoV-2 sub-lineages, exhibit genetic modifications/alterations with the potential to alter the virus’s epidemiological and clinical features; however, they lack confirmed public health implications, serving as early warning signals for health authorities to observe the evolutionary trajectory and potential future health implications.

#### Critical Bioinformatics Analysis of Mutational Mapping and Structural Overlap of RBD of the Recently Evolved NB.1.8.1 Sub-Variant (VUM)

**Figure 6:**
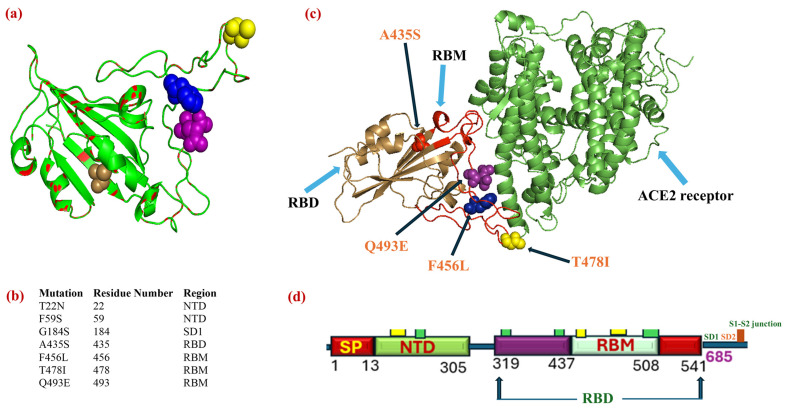
Depiction of mutational map of the most recently evolving SARS-CoV-2 VUM sub-variants (NB.1.8.1). (**a**) Superimposition (RMSD = 0.00) of mutated RBD of the NB.1.8.1 (red spots) and wildtype RBD (green) with three mutations in RBM and one mutation in RBD outside RBM (represented by sand color dots). (**b**) Key mutational features of SARS-CoV-2 VUM sub-variants (lineage-NB.1.8.1). (**c**) Representation of interaction of RBD with human ACE2 receptor and RBD mutations of sub-variants VUMs-NB.1.8.1. (**d**) S1-subunit of SASRS-CoV-2’s spike as well as genomic positions of several sub-domains of S1-subunit. Clusters of balls in different colors represent various mutations in those specific regions.

Following the spread of the SARS-CoV-2 lineage (JN.1), its sub-lineages (KP.3, KP.3.1.1, and XEC) evolved and spread worldwide. Later, LP.8.1 or JN.1.11.1.1.1.3.8.1 sub-variants descended from KP.1.1.3 or JN.1.11.1.1.1.3, which accounted for almost 30% of all infections worldwide as of the first four months of 2025 [173]. Most recently, the sub-variant NB.1.8.1 or XDV.1.5.1.1.8.1 with four RBD mutations (Figure 6a) and seven spike mutations in total (Figure 6b) has evolved and spread [179]. NB.1.8.1 descended from XDV. TXDV evolved as a recombinant lineage of XDE JN.1., whereas XDE is a recombinant lineage of GW.5.1 or XBB.1.19.1.5.1, as well as FL.13.4 or XBB.1.9.1.13.4. NB.1.8.1 with seven spike (S) mutations and twenty-three extra-spike mutations compared to JN.1 has emerged. NB.1.8.1 exhibits four RBD mutations (A435S, F556L, T478I, and Q493E) compared to the XEC spike protein [173] as mentioned in Table 3. The current research utilized bioinformatic tools, specifically the PyMOL Molecular Graphics System (Schrödinger, LLC), to visualize the genomic position of the spike mutations in the NB.1.8.1 sub-lineage.

Out of seven NB.1.8.1 spike substitutions, two mutations (T22N and F59S) are located in the N-terminal domain (NTD), one (G184S) in sub-domain 1, one (A435S) in RBD outside RBM, and three (F456L, T478I, and Q493E) in the RBM region. Moreover, using bioinformatics tools, the RBD of NB.1.8.1 was superimposed on the wildtype RBD to facilitate mutational mapping and structural overlap. The structural comparison between the wildtype SARS-CoV-2 RBD and the NB.1.8.1 RBD mutant was performed using PyMOL Molecular Graphics System (Version 3.1.6.1) (Schrödinger, LLC, New York, NY, USA). The crystallographic coordinates of the wildtype RBD were obtained from the Protein Data Bank, and point mutations were introduced directly through PyMOL scripting commands, rather than using the built-in mutagenesis wizard, to ensure precise control over residue substitution before structural alignment. The wildtype and mutant RBD structures were subsequently superimposed using backbone atoms, and the root mean square deviation (RMSD) was calculated to quantify conformational differences. The structural analysis of the NB.1.8.1 RBD mutant using PyMOL involved precise mutational mapping and structural alignment, providing valuable insights into the structural overlap and mutation impact. However, energy minimization or molecular dynamics simulations were not executed, which highlights the limitation of this study. Incorporating such validation techniques in future studies could further refine the understanding of the conformational changes.

An RMSD (root mean square deviation) of 0.00 Å between wildtype RBD (PDB ID: 6M0J) and NB.1.8.1 RBD suggested that the Cα atoms (or aligned atoms) are in identical spatial positions without considerable backbone shift, which indicates that the mutations possibly introduced only inside chains with no impact on the protein’s backbone. However, RMSD = 0.00 does not indicate that either the wildtype and mutated RBD of NB.1.8.1 are biologically identical, because RMSD does not account for side-chain orientation unless all the atoms are aligned.

**Figure 7:**
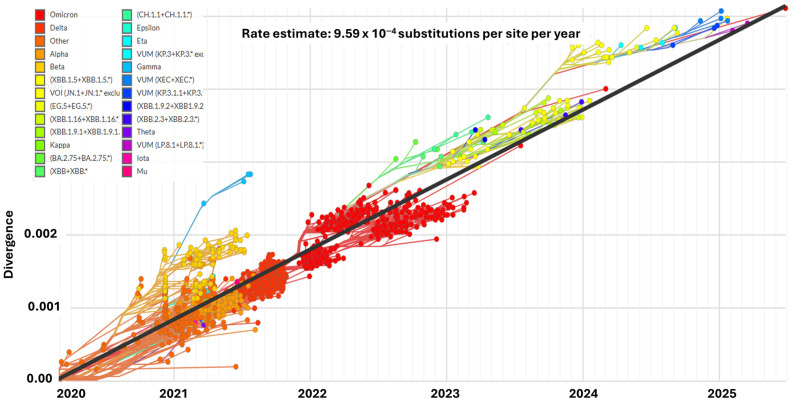
Temporal evolutionary divergence of SARS-CoV-2 variants from 2020 to 2025. The plot illustrates the progressive accumulation of mutations in SARS-CoV-2 genomes, expressed as divergence relative to the ancestral strain, across time. Distinct color-coded clusters represent major variants, including Alpha, Beta, Delta, and Omicron, along with their subsequent sub-lineages, variants of interest (VOIs), and variants under monitoring (VUMs). The black regression line indicates the estimated substitution rate of 9.59 × 10^−4^ substitutions per site per year. The figure highlights the accelerated diversification observed after the emergence of Omicron in late 2021, which gave rise to multiple sub-lineages that dominate the viral landscape into 2025 (https://gisaid.org/phylodynamics/angola/, accessed on 24 September 2025). Asterisk (*) denotes descendant sublineages.

Moreover, alterations such as Q493E, F456L, and T478I could affect binding and destabilize the interaction with the ACE2 receptor (Figure 6c), even if the backbone remains unchanged. Figure 6d represents the genomic positions of S1 subunit where these mutations occurred. Therefore, the current research recommends energy minimization, all-atom RMSD calculations, molecular dynamics simulation, and structural impact prediction analysis to gain a deeper insight into the biological impact of mutations in NB.1.8.1 RBD. Several blue spots are located on the RBM (residues ~437–508), consistent with known escape variants. RBM mutational hotspots, such as Q493E and T478I, could play a role in increasing ACE2 affinity and potential immune evasion, which corroborates the recent study’s findings that NB.1.8.1 exhibited higher ACE2 affinity and Ab-mediated evasion, indicating the potential of NB.1.8.1 for future dominance [180]. Compared with the current profile of BA.3.2, a variant with 50 mutations, NB.1.8.1. could outcompete the BA.3.2 dominance [173,180]. Therefore, sustained surveillance and monitoring of BA.3.2 and NB. 1.8.1 The evolutionary trajectory is necessary to assess the outbreak potential of these VUMs. The analysis was performed using GISAID data to track the temporal evolution of SARS-CoV-2 variants. The divergence was plotted and analyzed using color-coded clusters for each variant. Figure 7 illustrates the temporal evolutionary divergence of SARS-CoV-2 variants from 2020 to 2025 due to the accumulation of mutations (https://gisaid.org/phylodynamics/angola/, accessed on 24 September 2025).

Although emerging variants, such as Omicron variants, exhibit enhanced transmissibility and immune escape, current epidemiological evidence suggests that their virulence is generally reduced compared to earlier strains, such as Delta [181]. Thus, the evolution of SARS-CoV-2 favors higher transmissibility and persistence rather than uniformly increased virulence.

## 5. Conclusions

Over the years, the novel viruses that have evolved have contributed significantly to increasing infectiousness and the mortality rate. Advanced rapid diagnostic tools may be incapable of providing the initial and accurate diagnosis of the virus under study. The management and disease outcomes of the patients are greatly improved with the aid of molecular surveillance. However, extensive research work and publications are necessary to advance our knowledge about the evolutionary pattern of hCoVs and resolve many mysteries about the origination, possible scale, and modes of transmission, and the appropriate prevention as well as treatment of hCoVs. Additionally, the SARS-CoV-2-VOCs or SARS-CoV-2 VOIs or SARS-CoV-2 VUMs burden alters the SARS-CoV-2’s epidemiological and clinical features, poses extraordinary public health risks and preventive, diagnostic, and therapeutic challenges. Continuous surveillance/monitoring of continually emerging variants/sub-variants, especially VUMs, is paramount for scientists and health agencies in standardizing diagnostic accuracy, determining vaccine effectiveness, and prioritizing definitive therapeutic developmental research.

## Figures and Tables

**Table 1:** A comprehensive summary of critical mutations with their evolutionary and clinical implications of VOCs and VOIs.

VOCs or VOIs	Critical Mutations	Evolutionary and Clinical Implications	References
Alpha (α) (B.1.1.7 categorized as VOC). Gamma (γ) (P.1 categorized as VOC). Mu (VOI), Beta (β) (B.1.351/VOC). Omicron (VOC)	N501Y	Perform a major job during the first wave of VOCs. Higher transmissibility due to increased ACE2-receptor-RBD binding affinity. Vaccine protection was slightly diminished but remained effective, especially with a booster dose.	[134,135,136,137,138]
Alpha (α) (B.1.1.7 categorized as VOC), Omicron (B.1.1.529), Gamma (γ) grouped as VOC)	P681H	Increased transmissibility, infectivity, and severity. Enhancing protein cleavage by affecting furin-cleavage-site in the S-hot-spot, promoting virus-cell fusion and, therefore, viral entry into cells.	[136,139,140,141]
Alpha (α) (VOC), Gamma (γ) (P.1/VOC), Beta (β) (B.1.351/VOC), Mu (VOI)	E484K	Diminished binding affinity for neutralizing antibody. Remarkable immune escape and impact on vaccine protection. It impacts monoclonal therapy. Increases ACE2-receptor-RBD binding affinity.	[135,136,142,143]
Beta (β) also termed as B.1.351/VOC), Gamma (γ) categorized as VOC, Omicron (B.1.1.529), as well as Delta (δ) (VOC)	K417N	Enhanced ACE2-receptor-RBD binding affinity. Escape class 1 neutralizing antibody (nAb).	[135,136,143,144,145]
Gamma (γ) (P.1/VOC), Beta (β) (B.1.351/VOC), and Alpha (α)-sub-lineages.	L18F	Alter the configuration of the antigenic supersite in NTD. Evasion from NTD-specific nAbs.	[135,136,146,147]
Omicron (BA.2.75, XBB) and Delta (δ) (B.1.617.2/VOC)	T478K	It was part of a set of mutations triggering the spread of the Delta (δ) variant globally and persisted in Omicron variants/sub-variants, underscoring an evolutionary advantage. Aggrandize ACE2 binding and modify surface charge (electrostatic potential), possibly affecting antibody recognition and leading to evasion.	[147,148]
Delta (δ) (B.1.617.2/VOC)	P681R	cleavage of the S-protein into S1 and S2 by furin, leading to more efficient viral entry Facilitation of the furin-mediated spike protein cleavage, assisting viral fusion to the cell and potential entry.	[149]
Omicron (BA.4) as well as Delta (δ) (B.1.617.2 categorized as VOC)	L452R	Aggrandizes transmissibility and infectivity by stabilizing the ACE2-RBD interface. It was one of the clusters of mutations that fostered Delta’s greater transmissibility. Diminished Ab neutralization and downgraded cell-mediated immune response, allowing rapid viral replication	[150,151,152]
Lambda (γ) (VOI)	L542Q	Limited data are available. Slight immune evasion. Slight strengthening of the ACE2-RBD binding.	[153]
Lambda (γ) (C.37/VOI)	F490S	Alters receptor-binding motif (RBM) conformation, possibly affecting ACE2-Ab interaction, leading to immune evasion with nAb.	[154]
Lambda (VOI), alpha (α) (VOC), Beta (β) (VOC), Omicron (VOC), Gamma (γ) (VOC), Delta (δ) and (VOC)	D614G	It appeared in the initial state of the pandemic and became the most dominant mutation worldwide during the pandemic. It shows enhanced viral infectivity and replication. It enhances the transmission. It was observed to be allied with high viral load/increased infectivity, while the vaccine remained effective. Increased the S-protein’s open conformation stability and thus strengthened ACE2-RBD interaction. It was one of the major substitutions of the initial phase of the evolution.	[135,136,155]
Omicron (BA.1.1, BQ.1, and XBB.1.5/VOC) as well as Mu (μ) (B.1.621)/VOI)	R346K	Diminished neutralization by mAb and/or polyclonal sera.	[142]
Mu (B.1.621/VOI)	ins146N	Changes the closed–open S1-subunit conformation, fostering strengthened ACE2-RBD binding.	[142]
Sub-lineages evolved from Omicron (B.1.1.529/VOC)	G339D	Modify RBD’s local conformation and impact nAb binding and moderate evasion.	[144]
Omicron (B.1.1.529 categorized as VOC)	S477N	Aggrandized viral transmissibility (infectivity) via strengthening the ACE2-RBD interaction.	[156,157]
Omicron (BA.1, BA.2/VOC)	N440K	Enhanced viral fitness stabilizes ACE2-RBD binding. Foster resistance to mAb Evasion from natural or vaccine-induced nAb.	[144]
Omicron (BQ.1, BQ.1.1 sub-lineage, XBB.1.5, and XBB-sub-lineage, as well as CH.1.1/VOC)	R346T	Strongly allied with evasion from Class 3 mAb. Diminished Ab neutralization.	[158]
Omicron (XBB sub-lineage, XBB.1-sub-lineage, and XBB.1.5/VOC)	F486S	Enhanced neutralization of Abs (Class 1 & 2 nAb). Contributes to the increased fusogenicity.	[158]
Omicron (BA.1-sub-lineage and BA.2.75, as well as XBB-sub-lineage/VOC)	G446S	Foster resistance against Abs (Class 3 mAb and moderate evasion from polyclonal sera).	[158,159]
Omicron (BA.1/VOC)	R493Q	Evasion of Class 1 Ab. Enhances interaction with ACE2 receptor, easing adhesion to the cells	[158,159]

VOIs = Variants of Interest, VOCs = Varinats of Concern, and NTD = N-terminal Domain. nAbs = neutralizing antibodies, and mAbs = monoclonal antibodies.

**Table 2:** The genetic fingerprint of the VUMs originated from JN.1, with the next-strain clade information [166].

Pango-Lineage	Clade Information (Next-Strain)	Mutational-Fingerprint	The First Sampling Date	Variant Designation Date. Or the Risk Assessment Date	References ([166])
KP.2-sub-lineage also referred to as N.1.11.1.2	24C	Lineage-JN.1 + S:Q493E, S:F456L, S:V1104L, S:R346T	11 February 2024	3 May 2024	[160,167]
KP.3-sub-lineage or (JN.1.11.1.2)	24C	Lineage-JN.1 plus substitutions S:V1104L, S:F456L, S:Q493E,	11 February 2024	3 May 2024	[168]
KP.3.1.1 -sub-lineage or (JN.1.11.1.3.1.1)	24C	KP.3 + deletion S:S31-	27 March 2024	19 July 2024	[169]
LB.1 also referred to as JN.1.9.2.1	24A	Lineage-JN.1 + S:Q183H, S:R346T, S:F456L substitution, and one S:S31- deletion.	26 February 2024	28 June 2024	[170]
XEC	24F	JN.1 + S:F456L, S:T22N, S:F59S, S:V1104L, S:Q493E	26 June 2024	24 September 2024	[171]
LP.8.1	24B	JN1 + S:V445R, S:S31-, S:F186L, S:R346T, S:F456L, S:Q493E, S:V1104L, S:R190S, S:K1086R	1 July 2024	24 January 2025	[172]
NB.1.8.1	25B	JN1 + S:T22N, S:G184S, S:F59S, S:A435S, S:T478I, S:F456L, S:Q493E	22 January 2025	23 May 2025	[173]

**Table 3:** Comparison of spike and non-spike high frequency mutations in recently evolved SARS-CoV-2-lineage [173].

Genomic Region	SARS-CoV-2 Sub-Lineage (VUMs) as of May 2025					
	JN.1	KP.3	KP.3.1.1	XEC	LP.8.1	NB.1.8.1
N	G204R	G204R	G204R	G204P	G204R	G204R
	Q229K	Q229K	Q229K	Q229K	Q229K	-
ORF3	-	-	-	-	-	R138H
	-	-	-	-	P178L	-
NSP1	-	-	-	-	-	K47R
NSP2	-	-	-	-	-	S122F
	A31D	A31D	A31D	A31D	A31D	-
				A419T		-
NSP3	V238L	V238L	V238L	V238L	V238L	-
	-	-	-	-	-	A233Y
	-	-	-	-	-	A655V
	K1155R	K1155R	K1155R	K1155R	K1155R	-
	-	-	-	-	A1179V	-
	-	-	-	-	-	P1261Q
		T1465I	T1465I		T1465I	T1465I
	N1708S	N1708S	N1708S	N1708S	N1708S	
	-	-	-	-	-	I1891V
	A1892T	A1892T	A1892T	A1892T	A1892T	-
NSP4	-	-	-	-	-	L438F
NSP6	V24F	V24F	V24F	V24F	V24F	-
	R252K	R252K	R252K	R252K	R252K	-
NSP9	T35I	T35I	T35I	T35I	T35I	-
	-	-	-	-	-	P57S
	-	-	-	-	-	P80L
NSP10	-	-	S33C	-	-	-
NSP12	-	-	-	-	-	D284Y
	-	-	-	-	-	G671S
NSP13	-	-	-	-	-	S36P
S	-	-	-	T22N	-	T22N
	-	-	S31del	-	S31del	
	-	-	-	S59S		S59S
	-	-	-	-		G184S
	-	-	-	-	F186L	-
	-	-	-	-	R190S	-
	-	-	-	-	R346T	-
	-	-	-	-	-	A435S
	V455H	V455H	V455H	V455H	V455R	V455H
		F456L	F456L	F456L	F456L	F456L
	T478K	T478K	T478K	T478K	T478K	T478I
		Q493E	Q493E	Q493E	Q493E	Q493E
	-	-	-	-	K1086R	-
	-	V1104L	V1104L	V1104L	V1104L	-

## Data Availability

Data for this study are available in the manuscript in the form of figures and tables.
